# Supplementary material for: Head and Neck Squamous Cell Carcinoma Biopsies Maintained Ex Vivo on a Perfusion Device Show Gene Changes with Time and Clinically Relevant Doses of Irradiation
Source: Cancers (Basel). 2023 Sep 15;15(18):4575. doi: 10.3390/cancers15184575 (PMC10527562; doi:10.3390/cancers15184575)
Supplement: Supplementary file 1 [file cancers-15-04575-s001.zip › cancers-2599913-supplementary.pdf]

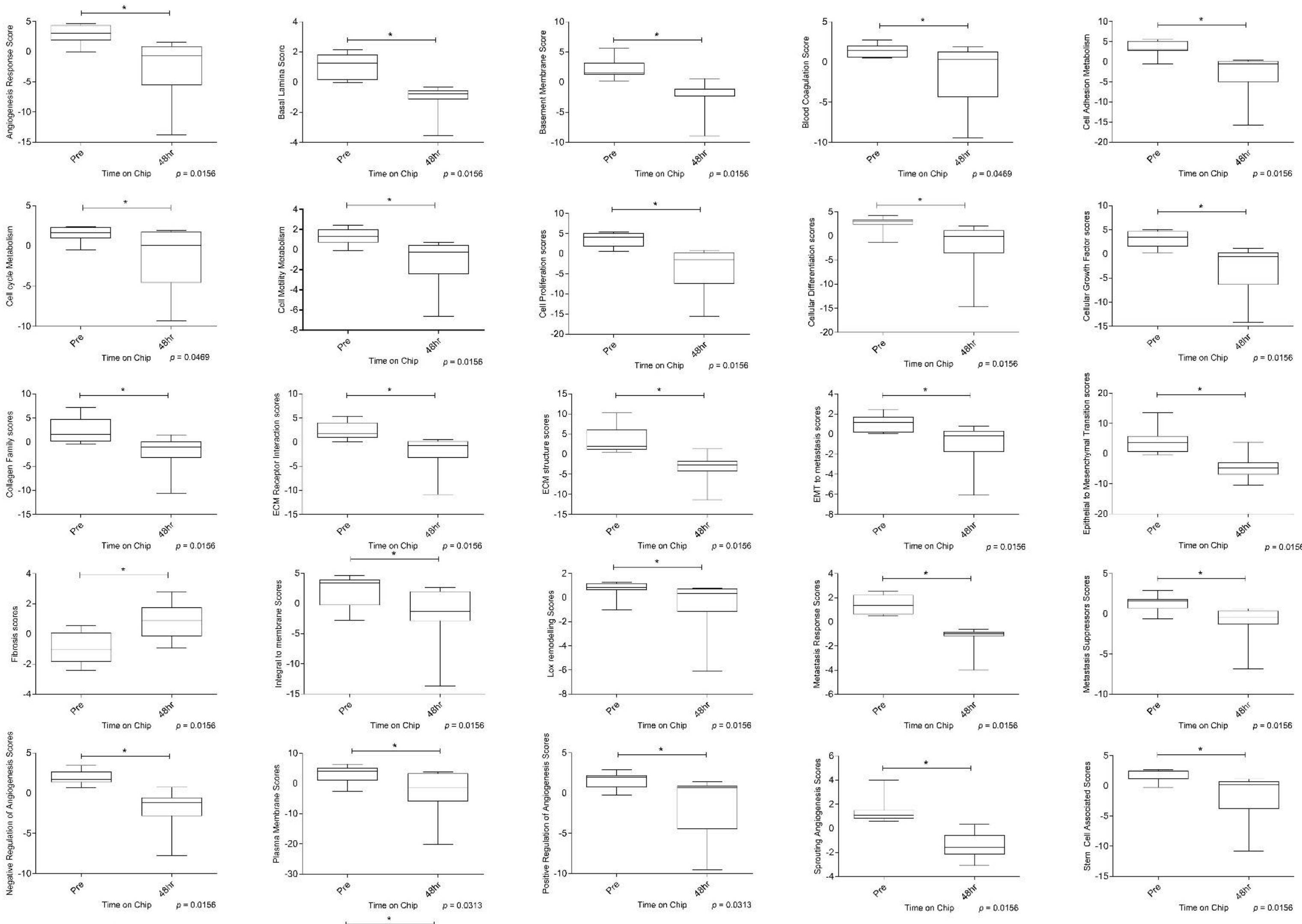

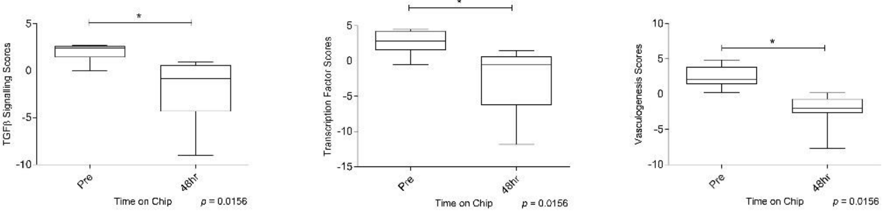

Figure S1. Box and Whisker plots showing pathways with significant differences in gene expression scores between the Pre samples and the samples incubated for 48hr on the perfusion device. *p<0.05 student t test

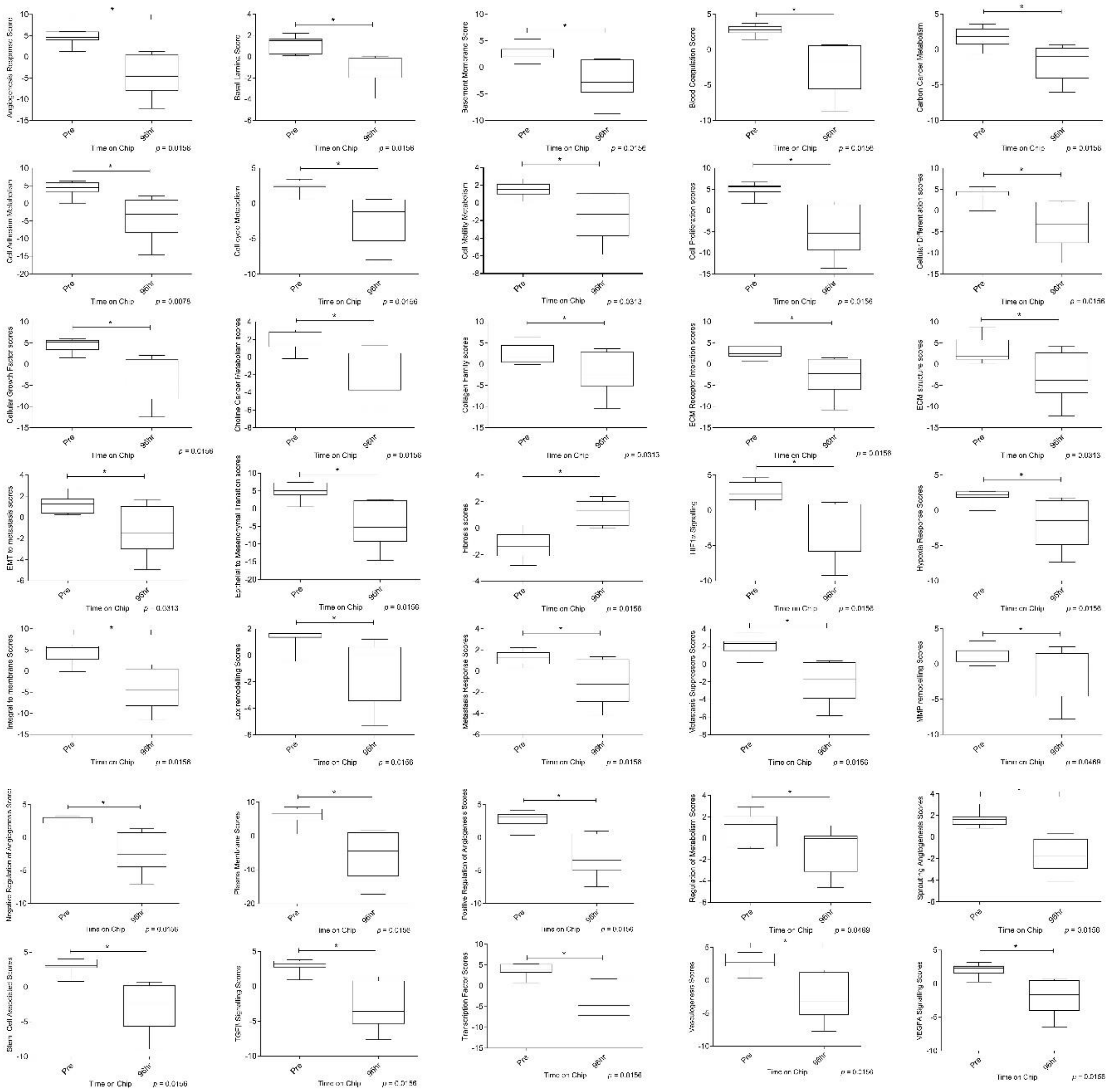

Figure S2. Box and Whisker plots showing pathways with significant differences in gene expression scores between the Pre samples and the samples incubated for 96hr on the perfusion device. *p<0.05 student t test
